# Longitudinal quantitative assessment of coronary atherosclerosis related to normal systolic blood pressure maintenance in the absence of established cardiovascular disease

**DOI:** 10.1002/clc.23870

**Published:** 2022-06-08

**Authors:** Ki‐Bum Won, Hyung‐Bok Park, Ran Heo, Byoung Kwon Lee, Fay Y. Lin, Martin Hadamitzky, Yong‐Jin Kim, Ji Min Sung, Edoardo Conte, Daniele Andreini, Gianluca Pontone, Matthew J. Budoff, Ilan Gottlieb, Eun Ju Chun, Filippo Cademartiri, Erica Maffei, Hugo Marques, Pedro de Araújo Gonçalves, Jonathon A. Leipsic, Sang‐Eun Lee, Sanghoon Shin, Jung Hyun Choi, Renu Virmani, Habib Samady, Kavitha Chinnaiyan, Daniel S. Berman, Jagat Narula, Jeroen J. Bax, James K. Min, Hyuk‐Jae Chang

**Affiliations:** ^1^ Department of Cardiology, Dongguk University Ilsan Hospital Dongguk University College of Medicine Goyang South Korea; ^2^ Department of Cardiology, Severance Cardiovascular Hospital, Yonsei University College of Medicine Yonsei University Health System Seoul South Korea; ^3^ Yonsei‐Cedars‐Sinai Integrative Cardiovascular Imaging Research Center, Yonsei University College of Medicine Yonsei University Health System Seoul South Korea; ^4^ Department of Cardiology Catholic Kwandong University International St. Mary's Hospital Incheon South Korea; ^5^ Department of Cardiology, Hanyang University Seoul Hospital Hanyang University College of Medicine Seoul South Korea; ^6^ Department of Cardiology, Gangnam Severance Hospital Yonsei University College of Medicine Seoul Korea; ^7^ Department of Radiology New York‐Presbyterian Hospital and Weill Cornell Medicine New York New York USA; ^8^ Department of Radiology and Nuclear Medicine German Heart Center Munich Munich Germany; ^9^ Division of Cardiology, Seoul National University College of Medicine, Cardiovascular Center Seoul National University Hospital Seoul South Korea; ^10^ Centro Cardiologico Monzino, IRCCS Milan Italy; ^11^ Department of Medicine Lundquist Institute at Harbor UCLA Medical Center Torrance California USA; ^12^ Department of Radiology Casa de Saude São Jose Rio de Janeiro Brazil; ^13^ Seoul National University Bundang Hospital Sungnam South Korea; ^14^ Cardiovascular Imaging Center, SDN IRCCS Naples Italy; ^15^ Department of Radiology Area Vasta 1/ASUR Marche Urbino Italy; ^16^ UNICA, Unit of Cardiovascular Imaging, Hospital da Luz Lisboa Portugal; ^17^ Nova Medical School Lisbon Portugal; ^18^ Department of Medicine and Radiology University of British Columbia Vancouver British Columbia Canada; ^19^ Department of Cardiology Ewha Womans University Seoul Hospital Seoul Korea; ^20^ Department of Cardiology Pusan University Hospital Busan South Korea; ^21^ Department of Pathology CVPath Institute Gaithersburg Maryland USA; ^22^ Department of Cardiology Emory University School of Medicine Atlanta Georgia USA; ^23^ Department of Cardiology William Beaumont Hospital Royal Oak Michigan USA; ^24^ Department of Imaging and Medicine Cedars Sinai Medical Center Los Angeles California USA; ^25^ Icahn School of Medicine at Mount Sinai, Mount Sinai Heart, Zena and Michael A. Wiener Cardiovascular Institute, and Marie‐Josée and Henry R. Kravis Center for Cardiovascular Health New York New York USA; ^26^ Department of Cardiology Leiden University Medical Center Leiden The Netherlands

**Keywords:** atherosclerosis, coronary artery disease, coronary computed tomography angiography, systolic blood pressure

## Abstract

**Background:**

Atherosclerosis‐related adverse events are commonly observed even in conditions with low cardiovascular (CV) risk. Longitudinal data regarding the association of normal systolic blood pressure maintenance (SBP_maintain_) with coronary plaque volume changes (PVC) has been limited in adults without traditional CV disease.

**Hypothesis:**

Normal SBP_maintain_ is important to attenuate coronary atherosclerosis progression in adults without baseline CV disease.

**Methods:**

We analyzed 95 adults (56.7 ± 8.5 years; 40.0% men) without baseline CV disease who underwent serial coronary computed tomographic angiography with mean 3.5 years of follow‐up. All participants were divided into two groups of normal SBP_maintain_ (follow‐up SBP < 120 mm Hg) and ≥elevated SBP_maintain_ (follow‐up SBP ≥ 120 mm Hg). Annualized PVC was defined as PVC divided by the interscan period.

**Results:**

Compared to participants with normal SBP_maintain_, those with ≥elevated SBP_maintain_ had higher annualized total PVC (mm^3^/year) (0.0 [0.0–2.2] vs. 4.1 [0.0–13.0]; *p* < .001). Baseline total plaque volume (*β* = .10) and the levels of SBP_maintain_ (*β* = .23) and follow‐up high‐density lipoprotein cholesterol (*β* = −0.28) were associated with annualized total PVC (all *p* < .05). The optimal cutoff of SBP_maintain_ for predicting plaque progression was 118.5 mm Hg (sensitivity: 78.2%, specificity: 62.5%; area under curve: 0.700; 95% confidence interval [CI]: 0.59–0.81; *p* < .05). SBP_maintain_ ≥ 118.5 mm Hg (odds ratio [OR]: 4.03; 95% CI: 1.51–10.75) and baseline total plaque volume (OR: 1.03; 95% CI: 1.01–1.06) independently influenced coronary plaque progression (all *p* < .05).

**Conclusion:**

Normal SBP_maintain_ is substantial to attenuate coronary atherosclerosis progression in conditions without established CV disease.

## 1 INTRODUCTION

Coronary atherosclerosis is strongly associated with an increase in cardiovascular (CV) morbidity and mortality.^1^, ^2^ CV risk has been stratified on the basis of conventional CV risk factors (CVRFs).^3^ However, atherosclerosis‐related adverse events are commonly developed even in adults with low CV risk.^4^, ^5^, ^6^ Considering that subclinical atherosclerosis underlies most CV events, it is important to identify independent predictors for subclinical atherosclerosis in conditions with a low CV risk burden.

The 2017 American College of Cardiology (ACC)/American Heart Association (AHA) guidelines lowered the blood pressure thresholds for defining hypertension.^7^ Although this enhanced guideline could cause overdiagnosis of hypertension and result in unnecessary treatment, recent studies have reported the usefulness of this guideline, especially in subjects with a low CV risk burden.^8^, ^9^ However, the consensus on this issue is not yet been reached in clinical practice. Recently, the Systolic Blood Pressure Intervention Trial (SPRINT) study finally reported that targeting a systolic blood pressure (SBP) of <120 mm Hg resulted in lower rates of major adverse CV events and mortality than targeting an SBP of <140 mm Hg in 9361 patients with an increased risk of CV disease who had no diabetes or previous stroke during a median of 3.3 years of follow‐up.^10^ Although this finding emphasizes the significance of strict SBP control with the consistent concept of reinforced guideline for hypertension, little is known about the optimal SBP levels to attenuate the progression of coronary atherosclerosis in conditions without established CV disease.

Serial assessment of coronary plaques using intravascular ultrasound (IVUS) has contributed to understanding the pathophysiology of coronary artery disease.^11^, ^12^ However, it is hard to perform IVUS in a low CV risk population because of its invasiveness and high cost. Recently improved technology in coronary computed tomographic angiography (CCTA) has allowed noninvasive evaluation and comprehension of coronary atherosclerosis.^13^, ^14^, ^15^, ^16^, ^17^, ^18^ Therefore, this study aimed to evaluate the association of normal SBP maintenance (SBP_maintain_) with coronary plaque volume changes (PVC) in adults without baseline CV disease using the quantitative measurement by serial CCTA.

## 2 RESEARCH DESIGN AND METHODS

### 2.1 study population

The study design and protocol of the Progression of AtheRosclerotic PlAque DetermIned by Computed TomoGraphic Angiography Imaging (PARADIGM) registry has been reported previously.^19^ Briefly, the PARADIGM is a prospective, international, and observational registry for evaluating associations of clinical factors with changes in coronary atherosclerosis using serial CCTA. Between 2003 and 2015, a total of 2252 consecutive participants underwent CCTA at 13 centers in seven countries. Of the 2252 participants, 1760 participants had an interpretable image quality by 0.5 mm cross‐sectional analysis following the Society of Cardiovascular Computed Tomography (SCCT) guidelines.^20^, ^21^ Among them, at index CCTA, 909 participants were identified as having no diabetes, and 814 participants were consecutively excluded because of the previous diagnosis of hypertension (*n* = 501), hyperlipidemia (*n* = 125), and atrial fibrillation (*n* = 23); obesity or unavailable body mass index (BMI) data (*n* = 10); current smoking (*n* = 53); any medication history (*n* = 91); and previous history of revascularization (*n* = 2) or cerebrovascular disease (*n* = 1), and unavailable follow‐up blood pressure data (*n* = 8). Finally, 95 participants without established CV disease at index CCTA were included in the present study. All participants were divided into two groups of normal SBP_maintain_ (follow‐up SBP < 120 mm Hg) (*n* = 40) and more than elevated SBP_maintain_ (follow‐up SBP ≥ 120 mm Hg) (*n* = 55) based on the follow‐up SBP levels, as per the 2017 ACC/AHA guidelines. Laboratory tests were conducted within 1 month of all CCTA examinations. All blood samples were collected after a minimum of 8 h fasting period. SBP and diastolic blood pressure (DBP) were measured on the right arm using an automatic manometer with an appropriate cuff size after the participants rested for ≥5 min.

### 2.2 acquisition and interpretation of CCTA

Data acquisition and post‐image processing were in accordance with SCCT guidelines.^20^, ^21^ All CCTAs were performed with a scanner with ≥ 64‐detector rows. Index and follow‐up datasets were transferred to an offline workstation for image analysis using semiautomated plaque analysis software (QAngioCT Research Edition v2.1.9.1; Medis Medical Imaging Systems) with manual correction.^22^ Independent level‐III experienced readers, blinded to clinical information, analyzed all CCTAs. Segments with a diameter of ≥2 mm were evaluated with a modified 17‐segment model.^20^, ^21^ Plaque volumes of every coronary segment were obtained and then summated to generate the total plaque volume on a per‐patient level. Atherosclerotic plaque volume was subclassified by composition, using predefined intensity cutoffs in Hounsfield units (HU) for calcified (≥351 HU), fibrous (131–350 HU), fibro‐fatty (31–130 HU), and necrotic‐core (−30–30 HU) that were validated.^23^, ^24^ For comparing atherosclerotic changes in CCTAs, both index and follow‐up coronary segments were registered together using fiduciary landmarks, including distance from ostia or branch vessel takeoffs. The PVC (mm^3^) was defined as the difference of plaque volume between the index and follow‐up CCTA at the per‐patient level. Annualized PVC (mm^3^/year) was defined as PVC divided by interscan period. Plaque progression was defined as the difference in plaque volume between follow‐up and index CCTA was more than zero. All methods were performed following the relevant guidelines and regulations. The appropriate institutional review board committees of Severance Cardiovascular Hospital approved the protocol of the present study.

### 2.3 statistical analysis

Continuous variables are expressed as mean ± SD or medians [interquartile range], as appropriate. Categorical variables are presented as absolute values and proportions. Continuous variables between two groups were compared using the independent *t*‐test or Mann–Whitney *U* test, as appropriate. Categorical variables were compared using the *χ* ^2^‐test or Fisher's exact test, as appropriate. Linear regression models were used to identify the association between clinical variables and annualized total PVC. Logistic regression models were used to identify the independent predictors of coronary plaque progression. Variables with *p* < .05 in the univariate analyses were considered confounding variables and entered into multivariate regression analyses, respectively. Except for the nonmodifiable variables of age, gender, and baseline plaque volume, other independent variables achieved at follow‐up CCTA were included in the regression models. In the receiver operating characteristic analysis, the optimal cutoffs of follow‐up SBP_maintain_ for predicting coronary plaque progression was determined using the Youden index. All statistical analyses were performed using the Statistical Package for the Social Sciences version 19 and SAS (version 9.1.3; SAS Institute Inc.). A *p* value of <.05 was considered significant for all analyses.

## 3 RESULTS

### 3.1 Baseline characteristics

The mean age of participants was 56.7 ± 8.5 years and the proportion of men was 40.0%. The mean interscan period was 3.5 ± 1.4 years. Table 1 presents the clinical characteristics of participants at both index and follow‐up CCTAs. Compared with participants with normal SBP_maintain_, those with ≥elevated SBP_maintain_ had significantly higher levels of SBP, DBP, and BMI at both CCTAs. No significant difference in statin use after index CCTA between participants with normal SBP_maintain_ and those with ≥elevated SBP_maintain_ was observed.

**Table 1:** Clinical characteristics

	Normal SBP_maintain_ (*N* = 40)	≥Elevated SBP_maintain_ (*N* = 55)	*p*
Age at enrollment, years	54.5 ± 8.1	58.4 ± 8.5	.028
Male, *n* (%)	11 (27.5)	27 (49.1)	.034
At index CCTA			
SBP, mm Hg	114.6 ± 14.1	123.9 ± 13.2	.003
DBP, mm Hg	71.7 ± 10.4	78.2 ± 9.4	.004
BMI, kg/m^2^	23.2 ± 2.8	24.5 ± 2.6	.025
Total cholesterol, mg/dL	189.3 ± 32.8	193.8 ± 34.2	.515
Triglyceride, mg/dL	109.8 ± 42.2	124.8 ± 70.9	.235
LDL‐C, mg/dL	116.6 ± 30.1	119.7 ± 27.8	.598
HDL‐C, mg/dL	55.9 ± 15.1	52.5 ± 13.1	.242
Glucose, mg/dL	94.4 ± 8.6	95.2 ± 9.7	.652
Hemoglobin A1C, %	5.7 ± 0.3	5.7 ± 0.3	.864
At follow‐up CCTA			
SBP, mm Hg	109.8 ± 6.1	126.8 ± 7.9	<.001
DBP, mm Hg	72.7 ± 7.3	77.8 ± 9.0	.004
BMI, kg/m^2^	23.1 ± 3.0	24.6 ± 2.4	.015
Total cholesterol, mg/dL	177.9 ± 37.1	185.6 ± 35.5	.305
Triglyceride, mg/dL	106.2 ± 42.9	112.2 ± 53.6	.563
LDL‐C, mg/dL	103.5 ± 33.3	112.1 ± 30.9	.195
HDL‐C, mg/dL	52.0 ± 11.0	51.2 ± 14.1	.758
Glucose, mg/dL	94.2 ± 11.3	97.2 ± 11.0	.201
Hemoglobin A1C, %	5.7 ± 0.3	5.8 ± 0.3	.219
Statin use, *n* (%)	14 (35.0)	12 (21.8)	.155

*Note*: Values are given as mean± standard deviation or number (%).

Abbreviations: BMI, body mass index; CCTA, coronary computed tomographic angiography; DBP, diastolic blood pressure; HDL‐C, high‐density lipoprotein cholesterol; LDL‐C, low‐density lipoprotein cholesterol; SBP, systolic blood pressure; SBP_maintain,_ systolic blood pressure maintenance.

### 3.2 Baseline and changes of coronary plaque volume

**Figure 1:**
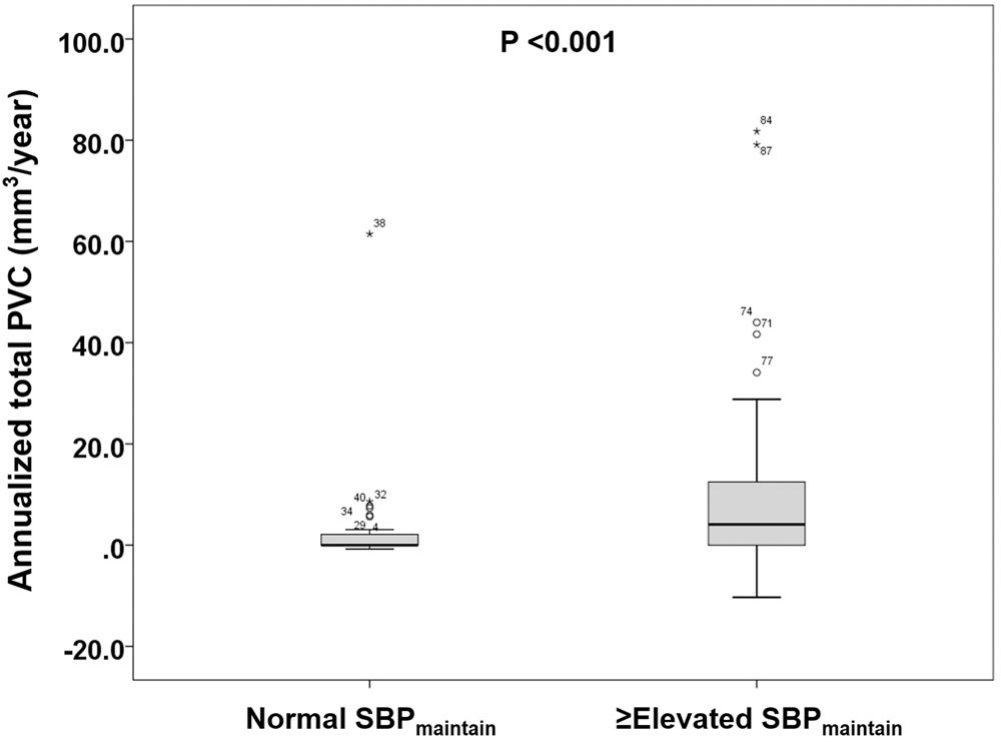
Comparison of annualized total PVC according to follow‐up SBP. PVC, plaque volume changes; SBP, systolic blood pressure; SBP_maintain,_ systolic blood pressure maintenance

Compared to participants with normal SBP_maintain_, the presence of plaque (35.5% vs. 60.0%; *p* = .016) and total plaque volume (mm^3^) (0.0 [0.0–16.0] vs. 11.1 [0.0–39.0]; *p* = .009) were higher in those with ≥elevated SBP_maintain_ at baseline (Table 2). Annualized total PVC (mm^3^/year) was lower in participantes with normal SBP_maintain_ than in those with ≥elevated SBP_maintain_ (0.0 [0.0–2.2] vs. 4.1 [0.0–13.0]; *p* < .001) (Figure 1). Regarding annualized PVC of coronary plaque subtypes, annualized dense calcium PVC, not annualized PVCs of fibrous, fibrous‐fatty, necrotic‐core, and dense calcium plaques, was significantly lower in participants with normal SBP_maintain_ than in those with ≥elevated SBP_maintain_ (0.0 [0.0–0.9] vs. 1.0 [0.0–4.2]; *p* < .001) (Table S1).

**Table 2:** Baseline coronary plaque characteristics

	Normal SBP_maintain_ (*N* = 40)	≥Elevated SBP_maintain_ (*N* = 55)	*p*
Baseline			
Presence of plaque, *n* (%)	14 (35.0)	33 (60.0)	.016
Total, mm^3^	0.0 [0.0–16.0]	11.1 [0.0–39.0]	.009
Fibrous, mm^3^	0.0 [0.0–7.8]	5.6 [0.0–22.9]	.011
Fibrous‐fatty, mm^3^	0.0 [0.0–0.4]	0.0 [0.0–12.1]	.010
Necrotic‐core, mm^3^	0.0 [0.0–0.0]	0.0 [0.0–0.4]	.051
Dense calcium, mm^3^	0.0 [0.0–1.7]	0.1 [0.0–8.4]	.020

*Note*: Continuous variables are given as medians [interquartile range].

Abbreviations: SBP, systolic blood pressure; SBP_maintain,_ systolic blood pressure maintenance.

### 3.3 Clinical variables and coronary PVC

Table 3 presents the results of linear regression models for the association between clinical variables and annualized total PVC. In the univariate linear regression analysis, baseline total plaque volume (per 1‐mm^3^ increase) (*β* = .11; *p* < .001), SBP_maintain_ (per‐1 mm Hg increase) (*β* = .38; *p* = .007) and follow‐up high‐density lipoprotein cholesterol (HDL‐C) (per 1‐mg/dL increase) (*β* = −0.34; *p* = .005) were associated with the annualized total PVC. The multivariate linear regression analysis showed that baseline total plaque volume (*β* = .10; *p* < .001), SBP_maintain_ (*β* = .23; *p* = .046), and follow‐up HDL‐C (*β* = −0.28; *p* = .008) were independently associated with the annualized total PVC.

**Table 3:** Association between clinical variables and annualized total PVC

	Univariate			Multivariate		
	*β*	95% CI	*p*	*β*	95% CI	*p*
Age at enrollment, years	.13	−0.24 to 0.49	.498			
Male	5.42	−0.80 to 11.63	.087			
Baseline total plaque volume, per ‐1 mm^3^ increase	.11	0.08–0.14	<.001	.10	0.07–0.13	<.001
At follow‐up						
BMI, per‐1 kg/m^2^ increase	.24	−0.95 to 1.43	.692			
SBP, per‐1 mm Hg increase	.38	0.11–0.65	.007	.23	0.01–0.45	.046
DBP, per‐1 mm Hg increase	.32	−0.03 to 0.68	.074			
Triglyceride, per ‐1 mg/dL increase	.07	0.01–0.13	.022	−.01	−0.07 to 0.05	.734
LDL‐C, per‐1 mg/dL increase	.06	−0.03 to 0.16	.191			
HDL‐C, per‐1 mg/dL increase	−.34	−0.56 to −0.11	.005	−.28	−0.48 to −0.07	.008
Glucose, per‐1 mg/dL increase	−.10	−0.38 to 0.18	.487			
Hemoglobin A1C, per‐1% increase	−1.34	−12.78 to 10.10	.815			
Statin use	4.20	−2.68 to 11.08	.228			

Abbreviations: BMI, body mass index; CI, confidence interval; DBP, diastolic blood pressure; HDL‐C, high‐density lipoprotein cholesterol; LDL‐C, low‐density lipoprotein cholesterol; PVC, plaque volume changes; SBP, systolic blood pressure.

### 3.4 Independent predictors for coronary plaque progression

The optimal SBP_maintain_ cut‐off for predicting coronary plaque progression was found to be 118.5 mm Hg, with 78.2% sensitivity and 62.5% specificity (area under curve: 0.70; 95% confidence interval [CI]: 0.59–0.81; *p* = .001) (Figure S1). In the univariate logistic regression analysis, male sex (odds ratio [OR]: 2.54; 95% CI: 1.06–6.08; *p* = .036), baseline plaque volume (per 1‐mm^3^ increase) (OR: 1.03; 95% CI: 1.01–1.06; *p* = .020), and SBP_maintain_ ≥ 118.5 mm Hg (OR: 5.97; 95% CI: 2.42–14.76; *p* < .001) were associated with plaque progression. The multivariate logistic regression analysis showed that baseline plaque volume (OR: 1.03; 95% CI: 1.01–1.06; *p* = .033) and SBP_maintain_ ≥ 118.5 mm Hg (OR: 4.03; 95% CI: 1.51–10.75; *p* = .005) independently influenced on plaque progression (Table 4).

**Table 4:** Independent predictors for coronary plaque progression

	Univariate			Multivariate		
	OR	95% CI	*p*	OR	95% CI	*p*
Age at enrollment, per‐1 year increase	1.04	0.99–1.09	.148			
Male	2.54	1.06–6.08	.036	2.03	0.74–5.56	.171
Baseline total plaque volume, per‐1 mm^3^ increase	1.03	1.01–1.06	.020	1.03	1.01–1.06	.033
At follow‐up						
SBP ≥ 118.5 mm Hg	5.97	2.42–14.76	<.001	4.03	1.51–10.75	.005
DBP, per‐1 mm Hg increase	1.05	0.99–1.10	.084			
BMI, per‐1 kg/m^2^ increase	1.14	0.97–1.34	.108			
Triglyceride, per‐1 mg/dL increase	1.01	0.99–1.02	.099			
LDL‐C, per‐1 mg/dL increase	1.01	0.99–1.02	.165			
HDL‐C, per 1‐mg/dL increase	0.98	0.95–1.01	.243			
Glucose, per 1‐mg/dL increase	1.00	0.96–1.04	.885			
Hemoglobin A1C, per 1% increase	1.45	0.30–7.02	.647			
Statin use	0.80	0.32–1.98	.624			

Abbreviations: BMI, body mass index; CI, confidence interval; HDL‐C, high‐density lipoprotein cholesterol; LDL‐C, low‐density lipoprotein cholesterol; OR, odds ratio; SBP, systolic blood pressure.

## 4 DISCUSSION

Data on the history of coronary atherosclerosis in subjects without established CV disease has been scarce because the performance of CCTA in this population is not yet justified despite the outstanding advances in CCTA technique. It was possible for the current study to evaluate this issue because PARADIGM is, to the best of our knowledge, the largest serial CCTA registry to date. The major findings of the present study were (1) SBP_maintain_, together with baseline total plaque volume, was independently associated with the progression of coronary atherosclerosis in subjects without established CV disease at baseline and (2) to maintain normal SBP levels was an important factor in attenuating coronary plaque progression in this population. Despite the characteristics of participants in the current study being completely different from those of the SPLINT study, these results showed the significance of normal SBP_maintain_ even in conditions of low CV risk burden. In addition, these findings could be substantial evidence to support the reinforced ACC/AHA guideline for hypertension.

Although the prevalence of hypertension definitely increases in clinical practice under the application of the 2017 ACC/AHA guideline,^7^ there has been a paucity of data on the usefulness of this blood pressure classification system, especially in the low CV risk population. Recently, Yano et al.^8^ reported that young adults aged <40 years with elevated blood pressure (hazard ratio [HR]: 1.67), Stage 1 hypertension (HR: 1.75), and Stage 2 hypertension (HR: 3.49), as defined by the 2017 ACC/AHA guidelines, had a significantly higher risk of subsequent CV events than those with normal blood pressure in the Coronary Artery Risk Development in Young Adults (CARDIA) study. Among East Asians, Liu et al.^9^ found that newly defined Stage 1 hypertension was associated with an increased risk of CV mortality in 154 407 Chinese adults, particularly among younger adults and those without a history of CV disease. Son et al.^25^ consistently reported that compared with normal blood pressure, Stage 1 and Stage 2 hypertension were associated with an increased risk of adverse CV events among 2 488 101 Korean adults aged 20–39 years. These studies strongly suggested that the reinforced 2017 ACC/AHA definition of hypertension may help to stratify CV risk in conditions with a low CV risk burden.

Recent studies have focused on the independent predictors for subclinical atherosclerosis in the absence of CVRFs.^26^, ^27^ The Progression of Early Subclinical Atherosclerosis (PESA) study reported that subclinical atherosclerosis, reflected in carotid, abdominal aortic, and iliofemoral plaques, and coronary artery calcification was present in one‐half of middle‐aged individuals without traditional CVRFs.^26^ However, although a significant difference in systolic blood pressure levels according to the presence of subclinical atherosclerosis was observed in PESA study, the association between systolic blood pressure and atherosclerosis was not assessed. Despite a novel concept on the predictors of subclinical atherosclerosis in the absence of CVRFs, little is known about the significance of maintianing normal SBP levels to attenuate coronary atherosclerosis progression in adults with low CV risk burden. In the present study, only 16.5% of participants were identified with SBP of Stage 1 and Stage 2 hypertension according to the ACC/AHA guideline at follow‐up. Hence, we categorized two groups as normal and more than elevated SBP_maintain_ in the present study. This study identified that the optimal cutoff of SBP_maintain_ for predicting coronary plaque progression was 118.5 mm Hg; this cutoff value might be influenced by the small number of newly developed cases of Stage 1 and Stage 2 hypertension during follow‐up periods. However, this result supports the rationality of reinforced ACC/AHA guideline for hypertension and reveals the significance of strict SBP control even in low CV risk status. Further investigation to assess the cost–benefit or cost‐effectiveness problems of strict SBP control as a primary preventive strategy should be necessary in clinical practice.

A recent experimental study has suggested that high blood pressure per se exacerbates atherogenesis of coronary arteries in the absence of proatherogenic humoral factors.^28^ In conditions without traditional risk factors such as hypertension, diabetes, hyperlipidemia, obesity, and current smoking, the present study found that annualized dense calcium PVC was significantly lower in participants with normal SBP_maintain_ than in those with ≥elevated SBP_maintain_. After adjusting for baseline volume of densely calcified plaque and statin use at follow‐up, normal SBP_maintain_ was related to the decreased risk of progression in dense calcified plaque (OR: 0.26; 95% CI: 0.10–0.68; *p* = .006). Considering the recent data showing that (1) the usefulness of coronary artery calcium score (CACS) to determine therapeutic targets in various clinical conditions^29^, ^30^, ^31^, ^32^ and (2) no significant prognostic benefit offered by CCTA when added to CACS and traditional risk factors in asymptomatic subjects,^33^ the CACS might be efficient tool to assess the progression of subclinical coronary atherosclerosis in conditions with low to intermediate CV risk burden.

Previous studies have evaluated the effect of specific antihypertensive agents on CV events and the progression of coronary atherosclerosis in patients with established coronary artery disease (CAD). The Comparison of Amlodipine vs Enalapril to Limit Occurrences of Thrombosis (CAMELOT) trial reported that the administration of amlodipine to patients with CAD and normal blood pressure resulted in reduced adverse CV events and attenuated atherosclerosis progression in 1991 patients with angiographically documented CAD and DBP < 100 mm Hg during a follow‐up of 24 months.^34^ This study provided a novel insight that the optimal blood pressure range for patients with CAD might be substantially lower than indicated by the guidelines. In the Impact of OLmesartan on the progression of coronary atherosclerosis: evaluation by IntraVascular UltraSound (OLIVUS) trial,^35^ the administration of olmesartan showed a positive role in a lower rate of coronary atherosclerosis progression in 247 stable angina pectoris patients with native CAD during a follow‐up of 14 month. On the contrary, the PERindopril's Prospective Effect on Coronary aTherosclerosis by IntraVascular ultrasound Evaluation (PERSPECTIVE) study found no beneficial effect on the progression of coronary atherosclerosis during 3.5 years of treatment with perindopril versus placebo as assessed with quantitative coronary angiography and IVUS in 244 patients with stable CAD who had no heart failure or substantial hypertension.^36^ The Aliskiren Quantitative Atherosclerosis Regression Intravascular UltrasoundStudy (AQUARIUS) showed that the use of aliskiren compared with placebo did not result in improving or slowing the coronary atherosclerosis progression after at least 72 weeks of randomization among 613 patients with CAD with prehypertension.^37^ Unlike the studies mentioned above, the present study identified the optimal SBP level for attenuating subclinical coronary atherosclerosis in conditions without established CV disease after adjusting for baseline plaque burden of coronary arteries which is known as the most important factor for rapid plaque progression.^38^

The present study has some limitations. First, consecutive changes of clinical variables during follow‐up periods were not available. Second, this study only included an extreme selection of participants from the PARADIGM registry who had no established CV disease. Therefore, the characteristics of our participants could not represent the overall participant characteristics of the PARADIGM registry. Third, the major proportion of the overall PARADIGM registry was East Asians. In this PARADIGM substudy, all participants were East Asians; hence, this might limit the generalizability of the findings. Fourth, the target blood pressure might be somewhat different in an elderly population.^39^ However, this study could not evaluate this issue because only five (4.9%) participants aged over 70 years. Finally, the small sample size and relatively short‐term follow‐up periods were the weaknesses of the present study. Despite these limitations, this is the first longitudinal study to evaluate the association of clinical factors with coronary atherosclerotic changes in the absence of established CV disease using serial CCTA.

In conclusion, compared to subjects with normal SBP_maintain_, annualized total PVC was significantly higher in subjects with ≥elevated SBP_maintain_ in the absence of baseline CV disease. Both baseline total plaque volume and SBP_maintain_ had an independent association with the risk of coronary plaque progression. The present study suggests that the endeavor to maintain normal SBP is important to attenuate coronary atherosclerosis progression even in conditions without established CV disease. Further prospective studies with larger sample sizes and longer follow‐up durations should be necessary.

## AUTHOR CONTRIBUTIONS

Ki‐Bum Won and Hyuk‐Jae Chang contributed to the conception and design of the work. Ki‐Bum Won, Hyung‐Bok Park, Ran Heo, Byoung Kwon Lee, Fay Y. Lin, Martin Hadamitzky, Yong‐Jin Kim., Ji Min Sung, Edoardo Conte, Daniele Andreini, Gianluca Pontone, Matthew J. Budoff, Ilan Gottlieb, Eun Ju Chun, Filippo Cademartiri, Erica Maffei, Hugo Marques, Pedro de Araújo Gonçalves, Jonathon A. Leipsic, Sang‐Eun Lee, Sanghoon Shin, Jung Hyun Choi, Renu Virmani, Habib Samady, Kavitha Chinnaiyan, Daniel S. Berman, Jagat Narula, Jeroen J. Bax, James K. Min, and Hyuk‐Jae Chang contributed to the acquisition, analysis, and interpretation of data. Ki‐Bum Won drafted the manuscript. Hyuk‐Jae Chang critically revised the manuscript. All authors gave final approval and agree to be accountable for all aspects of work ensuring integrity and accuracy.

## CONFLICTS OF INTEREST

Dr. James K. Min receives funding from the Dalio Foundation, National Institutes of Health, and GE Healthcare. Dr. Min serves on the scientific advisory board of Arineta and GE Healthcare, and has an equity interest in Cleerly. Dr. Habib Samaday has equity interest in Covanos. Dr. Leipsic serves as a consultant and has stock options in HeartFlow and Circle Cardiovascular Imaging; and receives grant support from GE Healthcare and speaking fees from Philips. All other authors declared no conflicts of interest.

## Supporting information


**Supplementary Material**.

**Supplementary figure 1**. Receiver operating characteristic curve of optimal SBP_maintain_ for predicting coronary plaque progression. SBP_maintain,_ systolic blood pressure maintenance.

**Supplementary table 1**. Changes of coronary plaque subtypes according to SBP_maintain_.
