# Supplementary material for: Longitudinal quantitative assessment of coronary atherosclerosis related to normal systolic blood pressure maintenance in the absence of established cardiovascular disease
Source: Clin Cardiol. 2022 Jun 8;45(8):873–81. doi: 10.1002/clc.23870 (PMC9346967; doi:10.1002/clc.23870)
Supplement: Supplementary file 1 — Supplementary Material. [file CLC-45-873-s001.docx]

**Supplementary appendix**

**Supplementary table 1.** Changes of coronary plaque subtypes according to SBP_maintain_

**Supplementary figure 1.** Receiver operating characteristic curve of optimal SBP_maintain_ for predicting coronary plaque progression

SBP_maintain,_ systolic blood pressure maintenance
