# Supplementary figures and images for: Longitudinal quantitative assessment of coronary atherosclerosis related to normal systolic blood pressure maintenance in the absence of established cardiovascular disease
Source: Clin Cardiol. 2022 Jun 8;45(8):873–81. doi: 10.1002/clc.23870 (PMC9346967; doi:10.1002/clc.23870)

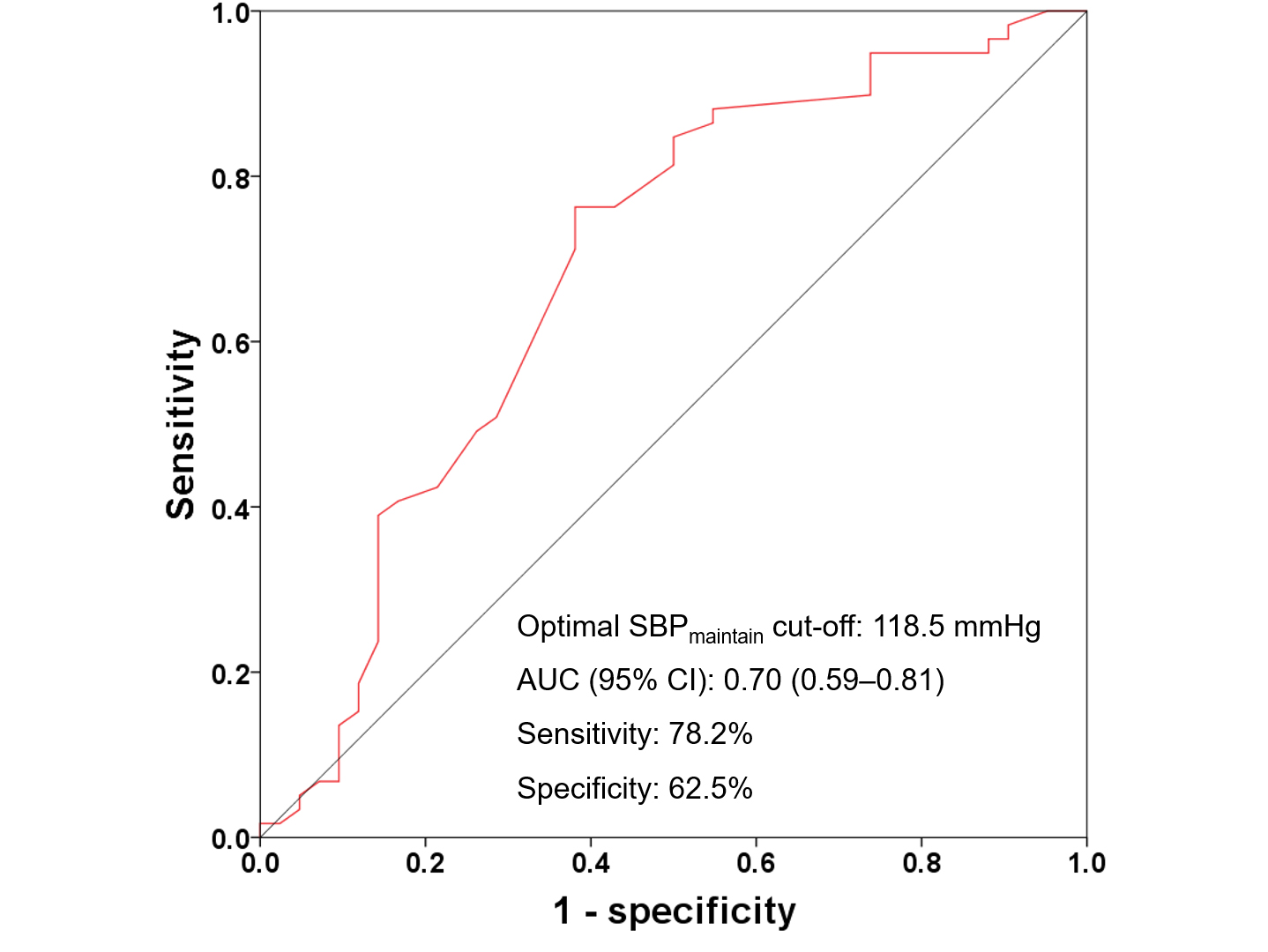

Supplement: Supplementary file 2 — Supplementary figure 1. Receiver operating characteristic curve of optimal SBPmaintain for predicting coronary plaque progression. SBPmaintain, systolic blood pressure maintenance. [file CLC-45-873-s003.jpg]
