# Supplementary material for: Longitudinal quantitative assessment of coronary atherosclerosis related to normal systolic blood pressure maintenance in the absence of established cardiovascular disease
Source: Clin Cardiol. 2022 Jun 8;45(8):873–81. doi: 10.1002/clc.23870 (PMC9346967; doi:10.1002/clc.23870)
Supplement: Supplementary file 3 — Supplementary table 1. Changes of coronary plaque subtypes according to SBPmaintain. [file CLC-45-873-s002.docx]

**Supplementary table 1.** Changes of coronary plaque subtypes according to SBP_maintain_

|  | Normal SBP_maintain_  (N = 40) | ≥elevated SBP_maintain_  (N = 55) | P |
| --- | --- | --- | --- |
| Annualized fibrous PVC, mm^3^/year | 0.0 [0.0–0.8] | 2.0 [0.0–7.0] | 0.062 |
| Annualized fibrous-fatty PVC, mm^3^/year | 0.0 [0.0–0.0] | 0.0 [0.0–2.2] | 0.317 |
| Annualized necrotic-core PVC, mm^3^/year | 0.0 [0.0–0.0] | 0.0 [0.0–0.0] | 0.766 |
| Annualized dense calcium PVC, mm^3^/year | 0.0 [0.0–0.9] | 1.0 [0.0–4.2] | <0.001 |

Continuous variables are given as medians [interquartile range].

PVC, plaque volume changes; SBP, systolic blood pressure; SBP_maintain,_ systolic blood pressure maintenance
